# Aggressive Approach in a Case of Cancer Cervix with Uremia

**DOI:** 10.4103/0973-1075.63136

**Published:** 2010

**Authors:** MG Janaki, S Mukesh, TR Arul Ponni, S Nirmala

**Affiliations:** Department of Radiation Oncology, M.S. Ramaiah Medical Teaching Hospital, Bangalore, India

**Keywords:** Carcinoma cervix, Chemotherapy, Radiotherapy, Renal failure

## Abstract

Carcinoma of cervix is the most common cancer in developing countries. Majority of them present in locally advanced stages. A 36-year-old lady presented with bleeding and white discharge per vagina since four months, vomiting and reduced urine output since two weeks. Patient had an exophytic cervical growth. Investigation revealed elevated serum creatinine. Patient received single fraction radiation and underwent percutaneous nephrostomy. At one month follow-up, serum creatinine returned to almost normal level. Patient underwent bilateral ante grade stenting and completed concurrent chemoradiotherapy. In selected subsets of patients, aggressive management offered longer palliation and good quality of life.

## INTRODUCTION

Cancer of the uterine cervix is one of the leading causes of cancer death among women worldwide. The estimated new cancer cervix cases per year are 500,000 of which 79% occur in the developing countries. Cancer cervix occupies either the top rank or second among cancers in women in the developing countries.[1] The cervical cancer burden in India alone is estimated as 100,000 in the year 2001. The differential pattern of cervical cancer and the wide variation in incidence are possibly related to environmental differences.[1] In urban areas, cancer of the cervix accounts for over 40% of cancers while in rural areas it accounts for 65% of cancers.[2] About 70% of them present as locally advanced disease, and one-third of them with renal failure.[2] Such patients have dismal prognosis and are usually managed with palliative radiation or sometimes best supportive care. We report the case of a patient who benefited from aggressive management.

## CASE REPORT

A lady aged 36 years presented with complaints of bleeding and white discharge per vagina since four months, vomiting and reduced urine output since two weeks. She also had anuria of eight hours duration. On examination, the patient had an exophytic cervical growth of eight centimeters, fitting into Federation Internationale de Gynecologie et d'Obstetrique (FIGO) Stage III B. Patient was investigated and found to have elevated serum creatinine (20.12 mg/dl), which had increased from 1.4 mg/dl over a period of one month. However, she did not have any other symptoms like pedal edema or facial puffiness and her electrolytes were normal. Ultrasound abdomen revealed bilateral hydronephrosis and an eight cm cervical growth and there was no pyometra. Biopsy showed a squamous carcinoma of Grade II variety. In view of renal failure the patient was given single fraction of 800 cGy to pelvis with palliative intent and she underwent left percutaneous nephrostomy. At one month follow-up, serum creatinine returned to almost normal level (1.4 mg/dl); PCN was removed and she underwent bilateral ante grade stenting. Patient completed concurrent radiotherapy to pelvis to a dose of 40 Gy/20 Fr/5 Fr/week with weekly carboplatin for five cycles and also received brachytherapy to a dose of 30 Gy to Point A. At 16^th^ month, the patient had no disease in the pelvis, however, she presented with pedal edema and icterus. Investigations revealed enlarged para aortic and node at common bile duct and dilated biliary radicals with a thrombus in the inferior vena cava. She succumbed to pulmonary embolism.

## DISCUSSION

Cervical cancer can spread to adjacent structures like the lower uterine segment, vagina and para cervical space along the broad and uterosacral ligaments. It can also have lymphatic and hematogenous spread. The parametrium is the connective tissue between the leaves of the broad ligament. Medially, it abuts the uterus, cervix, and proximal vagina. Laterally, it extends to the pelvic side wall. Inferiorly, it is contiguous with the cardinal ligament. The parametrium consists primarily of fat through which uterine vessels, nerves, fibrous tissues and lymphatic vessels run. The distal ureter is in the parametrium as it passes from the pelvic side wall to the bladder approximately two centimeters lateral to the margin of the cervix. When cervical cancer extends into the parametrium, the ureter can be encased by tumor and this leads to hydro ureteronephrosis and eventually renal failure.[3] Ureteral obstruction due to malignancy carries a poor prognosis with a resulting median survival of three to seven months[4] and hence most patients are treated with best supportive care or some palliative diversion procedure.

Such patients can be initially managed with ureteric stenting procedure or per cutaneous nephrostomy, to relieve the obstruction and then can be considered for chemo radiation. It is very important to select patients for curative treatment. Patients with features of uremia, frozen parametrium, non-functioning kidney are unlikely to show response and hence are best treated with supportive care.

According to Horan G *et al*. patients with bilateral hydronephrosis and a low (less than 50 mL/min (0.84 mL/s) creatinine clearance (CrCl) should be considered for elective stenting or per cutaneous nephrostomy prior to starting radiotherapy and pelvic radiation does not induce any deterioration of renal function or degree of hydronephrosis.[5] Similarly, Rotariu P *et al*. has concluded that placement of two double-J ureteral stents for the management of ureteral obstruction secondary to a malignancy is a safe and effective technique.[6]

The non-recovery of renal function after the relief of hydro ureteronephrosis is dependent on age and renal cortical thickness. Age beyond 50 years and decreased renal cortical thickness (less than 13 mm) indicate poor recovery of renal function. The complete renal recovery at 30^th^ day after diversion improves patient survival despite malignancy.[7] Our patient also had a normal creatinine at one month. Ishioka *et al*. have proposed a prognostic model based on the disease status, serum albumin and grade of hydronephrosis which predicts survival and hence helps in decision making for advanced, incurable malignancies.[8] Use of cisplatin as radio sensitizer with radiation is avoided as it is a nephrotoxic drug and worsens the pre existing renal failure. Various other chemotherapeutic drugs like carboplatin and gemcitabine can be tried. A study by Cetina *et al*. was conducted on nine patients with cervical cancer presenting with renal failure and treated with pelvic radiotherapy concurrently with weekly gemcitabine at 300 mg/m^2^. Four patients had a percutaneous nephrostomy placed and all patient completed chemoradiation. Toxicity was manageable. All but one patient had normalized serum creatinine. Eight (89%) of the nine patients achieved complete response and one patient had stable disease. At a median follow-up of 11 months, all patients were alive, one with pelvic and another with systemic disease.[9]

## CONCLUSION

All cases of locally advanced carcinoma cervix with ureteral obstruction causing varying degree of renal failure should not be considered as contraindication for concurrent chemo radiotherapy to attempt to cure. Aggressive management with an initial ureteric diversion may be curative in selected younger patients who have exophytic growth, less dense parametrial disease and who report early in the course of renal failure.
